# Cost-effectiveness of early inclisiran for the secondary prevention of cardiovascular disease in Aboriginal and Torres Strait Islander Australians: A Markov modelling analysis

**DOI:** 10.1016/j.ijcrp.2026.200687

**Published:** 2026-08-07

**Authors:** Satyen Hargovan, Nadine Hunt, Hara Kostakis, Clara K. Chow

**Affiliations:** a Department of Cardiology, The Prince Charles Hospital, Brisbane, Queensland, Australia; b College of Medicine and Dentistry, James Cook University, Queensland, Australia; c National Centre for Aboriginal and Torres Strait Islander Wellbeing Research and National Centre for Epidemiology and Population Health, College of Health and Medicine, The Australian National University, Australia; d Department of Public Health and Policy, London School of Hygiene and Tropical Medicine, University of London, UK; e Professor of Medicine, University of Sydney, NSW, Australia; f Consultant Cardiologist, Westmead Hospital, Sydney, NSW, Australia; g Academic Director, Westmead Applied Research Centre, University of Sydney, NSW, Australia

## Abstract

**Background:**

Aboriginal and Torres Strait Islander Australians experience substantially higher rates of cardiovascular disease (CVD), premature mortality, and related healthcare expenditure than non-Indigenous Australians. Improved management of modifiable risk factors, including hypercholesterolaemia, may reduce this excess burden. Inclisiran is a small interfering RNA therapy that provides sustained reductions in low-density lipoprotein cholesterol (LDL-C). Its cost-effectiveness as an earlier secondary prevention strategy in high-risk populations remains uncertain. We evaluated the cost-effectiveness of earlier addition of inclisiran to statin therapy for Aboriginal and Torres Strait Islander Australians with established CVD.

**Methods:**

A health economic analysis was undertaken from the Australian healthcare system perspective. A 25-year Markov cohort state-transition model with annual cycles was developed using the best available Australian epidemiological and cost data. Aboriginal and Torres Strait Islander Australians with established CVD and hypercholesterolaemia were modelled to receive inclisiran plus statin therapy or statin therapy alone. Model outputs included the incremental cost-effectiveness ratio (ICER), non-fatal CVD events avoided, and quality-adjusted life years (QALYs) gained. Deterministic sensitivity analyses were performed.

**Results:**

Among an estimated 12,180 eligible individuals, earlier inclisiran use was modelled to avert 6401 non-fatal CVD events and gain 23,730 QALYs over 25 years, at an additional cost of AUD 1.16 billion (AUD 46.3 million annually; 0.32% of annual Australian CVD expenditure). The ICER was AUD 48,808-per-QALY gained. Cost-effectiveness improved with lower inclisiran pricing and earlier treatment initiation.

**Conclusions:**

In this modelled analysis, earlier addition of inclisiran to statin therapy may represent a cost-effective secondary prevention strategy for Aboriginal and Torres Strait Islander Australians with established CVD. Earlier access could reduce projected cardiovascular burden and may inform future reimbursement, guideline, and equity-focused prevention policy in Australia.

## Highlights

• Aboriginal and Torres Strait Islander Australians bear disproportionate CVD risk.
• Twice-yearly inclisiran enables potent LDL-C lowering with possible improved adherence.
• Early inclisiran was modelled to avert 6401 non-fatal CVD events and gain 23,730 QALY's.
• Modelled ICER was AUD48,808/QALY; equivalent to 0.32% of annual CVD spend.
• Earlier access may support more equitable cardiovascular prevention strategies.

## 1 Introduction

Reducing inequities in cardiovascular outcomes remains a major public health priority. In Australia, the national Closing the Gap strategy seeks to eliminate disparities in life expectancy between Aboriginal and Torres Strait Islander Australians and non-Indigenous Australians [1]. Despite this commitment, Aboriginal and Torres Strait Islander Australians continue to experience a substantially higher burden of cardiovascular disease (CVD), with excess premature mortality, recurrent hospitalisation, disability, and healthcare expenditure [2]. CVD remains one of the leading contributors to the persistent life expectancy gap, highlighting the need for more effective and scalable preventive strategies.

Hypercholesterolaemia is a major modifiable driver of atherosclerotic cardiovascular disease (ASCVD). The Australian Institute of Health and Welfare has estimated that the population attributable fraction of hypercholesterolaemia to CVD risk among Aboriginal and Torres Strait Islander Australians is 29% [2]. Elevated low-density lipoprotein cholesterol (LDL-C) is causally associated with ASCVD, and extensive evidence demonstrates that lowering LDL-C proportionally reduces major adverse cardiovascular events [3]. Accordingly, contemporary international guidelines consistently recommend early and intensive LDL-C lowering, particularly in secondary prevention populations [3].

Although efficacious at reducing LDL-C and MACE, the utility of first-line lipid-lowering statins and second-line lipid-lowering Ezetimibe are impeded by adherence, pill-burden, adverse effects, clinical inertia and prescribing barriers [4]. Subsequently, many patients remain at-risk, particularly Aboriginal and Torres Strait Islander Australians who have well-defined decreased access to, and utilisation of, healthcare resources despite increased need driven by the social determinants of health [5].

Inclisiran, a Proprotein Convertase Subtilisin–kexin Type 9 (PCSK9) Small-interfering Ribonucleic Acid (siRNA), offers rapid, safe, durable and tolerable LDL-C reduction [6]. The recent VICTORION-INITIATE trial demonstrated improved LDL-C therapeutic goal attainment and lower risk of MACE with earlier inclisiran initiation [7]. A further unique differentiatior over other lipid-lowering therapies is its’ 6-monthly dosing, which reduces pill-burden, improves adherence and reduces clinical inertia to LDL-C lowering in real-world studies which may ultimately reduce MACE [4,8]. Whilst subsidised by the Australian governments Pharmaceutical Benefits Advisory Committee (PBAC) for patients with CVD and hypercholesterolaemia, inclisiran remains distant third-line lipid-lowering therapy subject to meeting multiple onerous strict criteria thereby limiting access for high-risk patients who may derive the greatest absolute benefit (Table 1) [9].

**Table 1:** Current PBS indication for Inclisiran compared to our proposed indications.

Current PBS indications for Inclisiran	Our proposed strategy for Inclisiran
Symptomatic CVD AND trialled diet/exercise AND trialled high-dose Statin for 3 months (or developed intolerance) AND trialled Ezetimibe for 3 months (or developed intolerance) AND have either: • Atherosclerotic-CVD in >2 vascular territories OR • 2 CVD events in the last 5 years OR • Diabetes with microalbuminuria OR • Diabetes and >60 years old OR • Aboriginal and Torres Strait Islander Australian with diabetes OR • TIMI (thrombolysis in Myocardial Infarction) score of >4 AND LDL-C still >1.8 mmol/L	Early, first-line with standard Statin therapy IF: Aboriginal and Torres Strait Islander Australian AND diagnosed with atherosclerotic CVD AND LDL-C >1.8 mmol/L

Whether earlier use of inclisiran could represent a clinically effective and economically efficient strategy for populations with disproportionate cardiovascular risk remains unknown. We therefore evaluated the cost-effectiveness of early addition of inclisiran to statin therapy for secondary prevention in Aboriginal and Torres Strait Islander Australians with established CVD and hypercholesterolaemia, using a long-term Markov model from the Australian healthcare system perspective. We hypothesised that an earlier, equity-focused treatment strategy may reduce cardiovascular burden and provide value-based guidance for future preventive cardiology policy.

## 2 Methods

### 2.1 Ethics and planning

The study was designed in consultation with multidisciplinary team members, including Indigenous Liaison Officers from the Better Cardiac Care and Aboriginal and Torres Strait Islander Cardiology Outreach services at Cairns Hospital, and was informed by the Australian Institute of Aboriginal and Torres Strait Islander Studies (AIATSIS) Code of Ethics for Aboriginal and Torres Strait Islander Research. An Aboriginal and Torres Strait Islander co-author (Iamalaig and Kaantju woman; National Centre for Aboriginal and Torres Strait Islander Wellbeing Research, Australian National University) contributed to culturally informed study design, interpretation, and framing. Ethics approval was obtained from the London School of Hygiene and Tropical Medicine (approval no. 28464) and the James Cook University Human Research Ethics Committee, including Aboriginal and Torres Strait Islander ethics subcommittee approval (24H-9669).

### 2.2 Setting and design

**Fig. 1:**
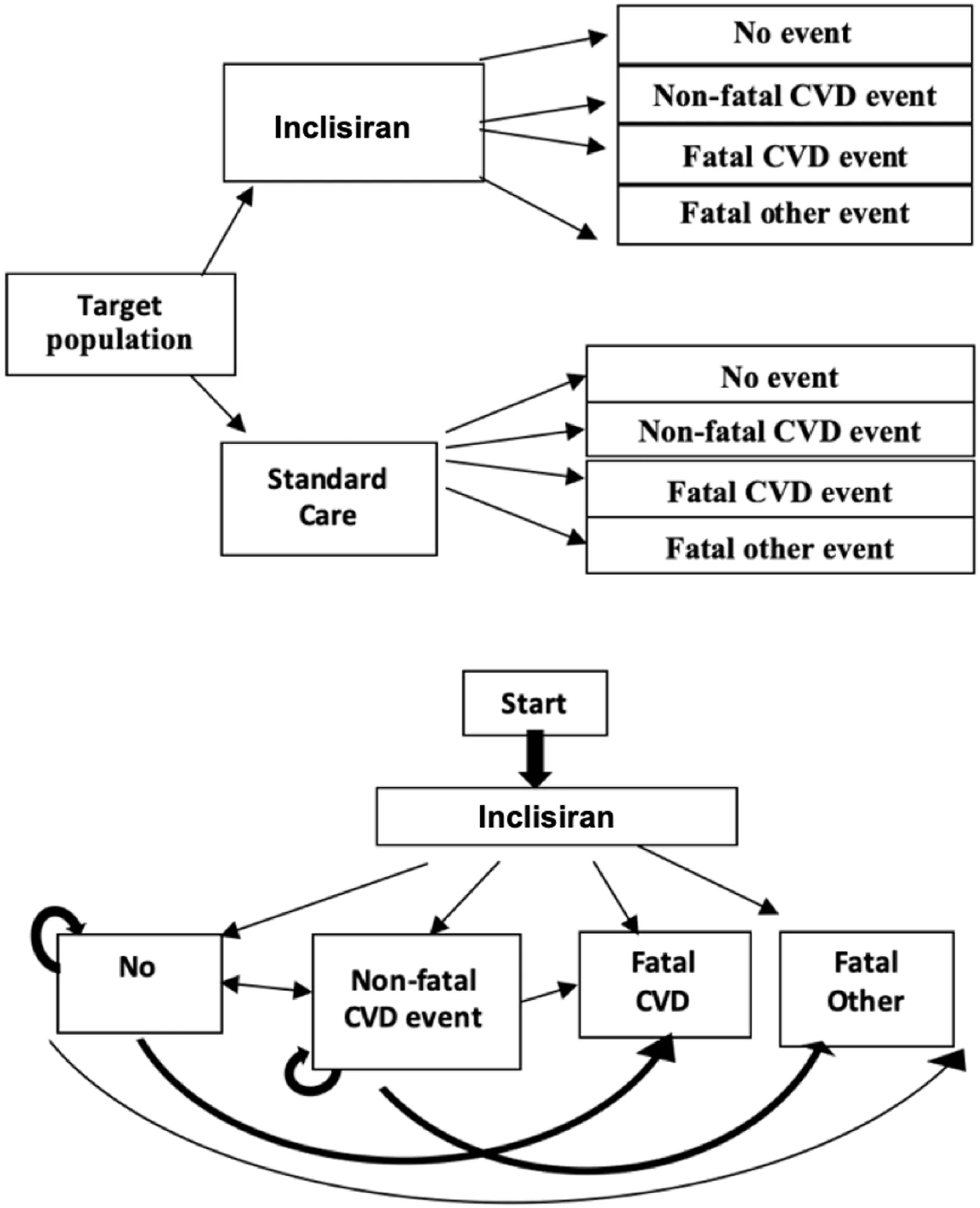
Proposed Markov Model health states and transitions.

A cohort state-transition Markov model with annual cycles was constructed using Microsoft Excel (Microsoft Corporation, USA) (Table 2, appendix 1). This is similar in structure to previously published peer-reviewed Australian cardiovascular economic models in Aboriginal and Torres Strait Islander populations [10]. The population, defined in Table 3, were Aboriginal and Torres Strait Islander Australians with CVD and hypercholesterolaemia based on AIHW estimates. The perspective taken was that of the Australian Healthcare system. The intervention was inclisiran with Statin. The comparator was current first-line PBS funded, guideline-directed Australian standard-of-care (ie, statins alone). Ezetimibe's real-world uptake is estimated at <10% in this population and was therefore not explicitly modelled as a separate treatment pathway [6]. As no data exists on the real-world use of inclisiran in Aboriginal and Torres Strait Islander Australians, adherence was assumed at 100% across both arms to isolate pharmacological treatment effect and avoid confounding from differential adherence assumptions. The modelled outcomes were standard no event, fatal CVD event, non-fatal CVD event or fatal-other event. Time was cycled annually to 25 years (the difference between average Aboriginal and Torres Strait Islander Australian life expectancy and first CVD event). The primary outcome was the Incremental cost-effectiveness ratio (ICER) which reflected cost-per-quality-adjusted life year (QALY) gained. QALYs were used to align with conventions in healthcare economic evaluation reporting. Secondary outcomes were CVD events and QALY's gained. Proposed health states and transitions are defined in Fig. 1.

**Table 2:** Summary table of Markov model input parameters.

Intervention	Model variables	Health state	Sensitivity Range	Source:	Values:	Distribution:
Early Inclisiran with Standard Therapy	Annual Transition Probabilities	No event	±5%		61.11%	Beta
		Non-fatal CVD event	±5%	Pooled ORION 9/10/11 trials	36.02%	Beta
		Fatal CVD event	±5%	Pooled ORION 9/10/11 trials	2.85%	Beta
		Fatal other event	±5%		1.54%	Beta
	Utility weights	No event	±5%	VALIANT trials	0.8	Beta
		Non-fatal CVD event	±5%	VALIANT trials	0.7	Beta
		Fatal CVD event and other events	±5%		0	Beta
	Cost Parameters	Statin + Inclisiran	±5%	PBS	$3922	Gamma
		No event	±5%	Australian DRG data	$4878	Gamma
		Non-fatal CVD event	±5%	Australian DRG data	$21,943	Gamma
		Fatal CVD event	±5%	Australian DRG data	$76,927	Gamma
		Fatal Other event	±5%	Australian DRG data	$76,927	Gamma
Standard Therapy	Annual Transition Probabilities	No event	±5%		57.33%	Beta
		Non-fatal CVD event	±5%	Australian Indigenous Burden of Disease study	38.39%	Beta
		Fatal CVD event	±5%	Australian Indigenous Burden of Disease study	2.74%	Beta
		Fatal other event	±5%	Australian Indigenous Population Statistics	1.54%	Beta
	Utility weights and Cost parameters	Same as Inclisiran arm

**Table 3:** Estimated annual burden of CVD attributed to hypercholesterolaemia in Aboriginal and Torres Strait Islander Australians with CVD.

Cohort	Number at-risk	CVD-related hospitalisations	CVD-related deaths	CVD-related DALY's	Annual CVD Healthcare costs (AUD)
Aboriginal and Torres Strait Islander Australians with CVD	42,000	17,275	1150	24,612	$424,800,000
Aboriginal and Torres Strait Islander Australians with CVD attributable to Hypercholesterolaemia (target population)	12,180	5010	334	7.137	$123,192,000

### 2.3 Transition probabilities – intervention arm

Model inputs for lipid-lowering efficacy and cardiovascular events were derived from pooled patient-level analyses of the ORION 9/10/11 RCT's [6]. These observed trial data were subsequently extrapolated within a validated Markov framework to estimate long-term cardiovascular outcomes, QALYs, and costs. Long-term projections therefore represent modelled estimates beyond the duration of trial follow-up. Similar methodology has been used in similar peer-reviewed Australian inclisiran cost-effectiveness study for non-Aboriginal and Torres Strait Islander Australians [11]. The inclisiran arm had 17 fatal CVD events in 1833 patients (0.93%) versus placebo arm (15/1827, 0.82%); an absolute risk increase of 0.11%. The inclisiran arm had 114 non-fatal CVD events (6.22%) versus placebo arm (157/1827, 8.59%); an absolute risk reduction (ARR) of 2.37% [6]. These estimates were used to inform transition probabilities. Importantly, these trial-derived estimates were applied as relative risk inputs rather than assumed absolute effects in the base model to preserve consistency with modelling conventions. MACE definitions were aligned with ORION trials to ensure consistency [6].

### 2.4 Transition probabilities – comparator arm

Aboriginal and Torres Strait Islander Australian's with CVD and hypercholesterolaemia have no published transition probability data. Best available national administrative and epidemiological data from Australian Government sources were used as calibrated population-level proxies as presented in Table 3 (data accessed between August-October 2024).

Of the 3.2% (812,728) total Aboriginal and Torres Strait Islander Australians [11], approximately 42,000 have known CVD accounting for 17,275 CVD-related hospitalisations, 1150 CVD-related deaths, 24,612 CVD-related QALY's gained [12], and 3.6% of the total $11.8 billion Australian CVD healthcare expenditure ($424,800,000) annually [1]. The AIHW estimates the population attributable fraction of hypercholesterolaemia to this CVD burden in Aboriginal and Torres Strait Islander Australians is 29% [1]. Thus, the baseline population entering our model were the estimated 12,180 Aboriginal and Torres Strait Islander Australians with CVD and hypercholesterolaemia of whom 334 experience fatal CVD events and 4676 non-fatal CVD events yearly (Table 3). The fatal-other event rate reflected age-adjusted mortality rates and was held constant between arms to avoid competing risk bias and isolate cardiovascular-specific effects^.^ [11].

### 2.5 Costs and discounting

All costs were reported in 2025 Australian dollars (AUD). Annual statin therapy costs were estimated at AUD 223 per person using weighted average generic pricing [13]. Annual inclisiran acquisition cost was AUD 3699 per person (July 2025 pricing [14]. Costs associated with no event, non-fatal CVD events, and fatal CVD events were derived from Australian diagnosis-related group (DRG) funding data and aligned with previous Australian cardiovascular economic evaluations [11,14]. The Pharmaceutical Benefits Advisory Committee (PBAC) has no defined willingness-to-pay threshold (WTPT) to determine whether an intervention is cost-effective. Whilst other factors including clinical need and equity are also considered, generally <$50,000AUD/QALY WTPT is commonly used as a reference benchmark in Australian health economic evaluations [15]. Costs and outcomes were discounted at 5% per annum.

### 2.6 Utilities

No published health-state utility estimates were identified specifically for Aboriginal and Torres Strait Islander Australians with established CVD and hypercholesterolaemia. Utility estimates were therefore informed by the VALIANT cohort and prior Australian cardiovascular models [11,14,16]. Utility values were 0.80 for established CVD without recurrent event and 0.70 following non-fatal recurrent CVD events. Utility values for cardiovascular and non-cardiovascular death were 0.

### 2.7 Sensitivity analysis

Deterministic one-way sensitivity analyses were performed to assess model robustness. Key parameters varied included inclisiran cost, treatment duration, discount rate, utility estimates, comparator event rates, and treatment-effect assumptions. Unless otherwise specified, parameters were varied by ±5%. As robust variance estimates were unavailable for several key model inputs, broader (±20%) scenario analyses were additionally performed for the principal model drivers to further explore uncertainty in the absence of probabilistic sensitivity analysis. Probabilistic sensitivity analysis was not performed because robust variance estimates were unavailable for several key population-level parameters derived from administrative and epidemiological datasets.

### 2.8 Model validation

Face validity was assessed through clinical review by cardiovascular and Indigenous health experts involved in study design with knowledge in healthcare economic analysis. The systematic reporting on validation efforts for our Markov model are presented via the Assessment of the Validation Status of Health-Economic decision models (AdViSHE) tool (appendix 2) [17].

## 3 Results

### 3.1 25-Year Markov Model

**Fig. 2:**
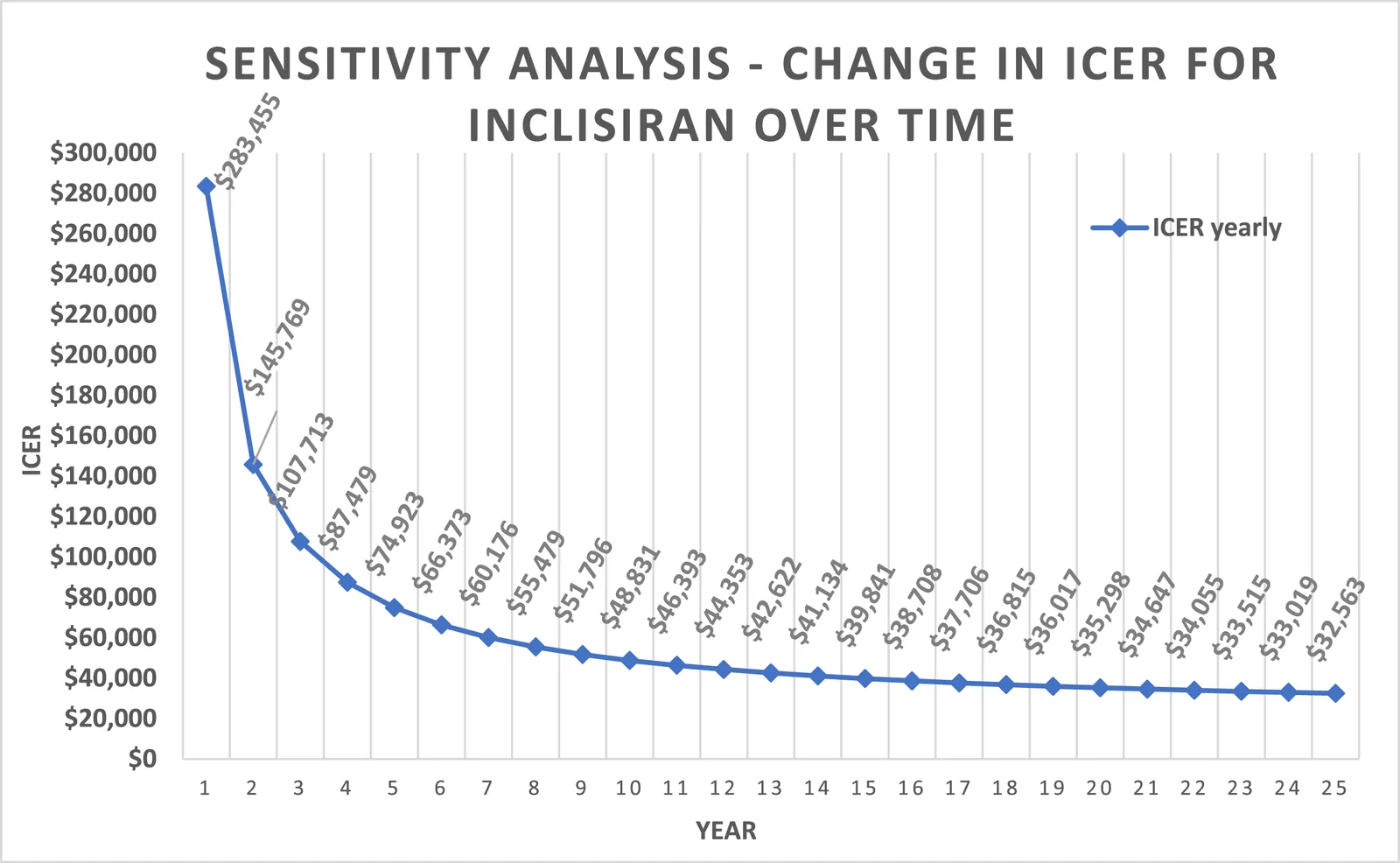
Sensitivity analysis – Yearly change in ICER per QALY for Inclisiran over 25-years.

**Fig. 3:**
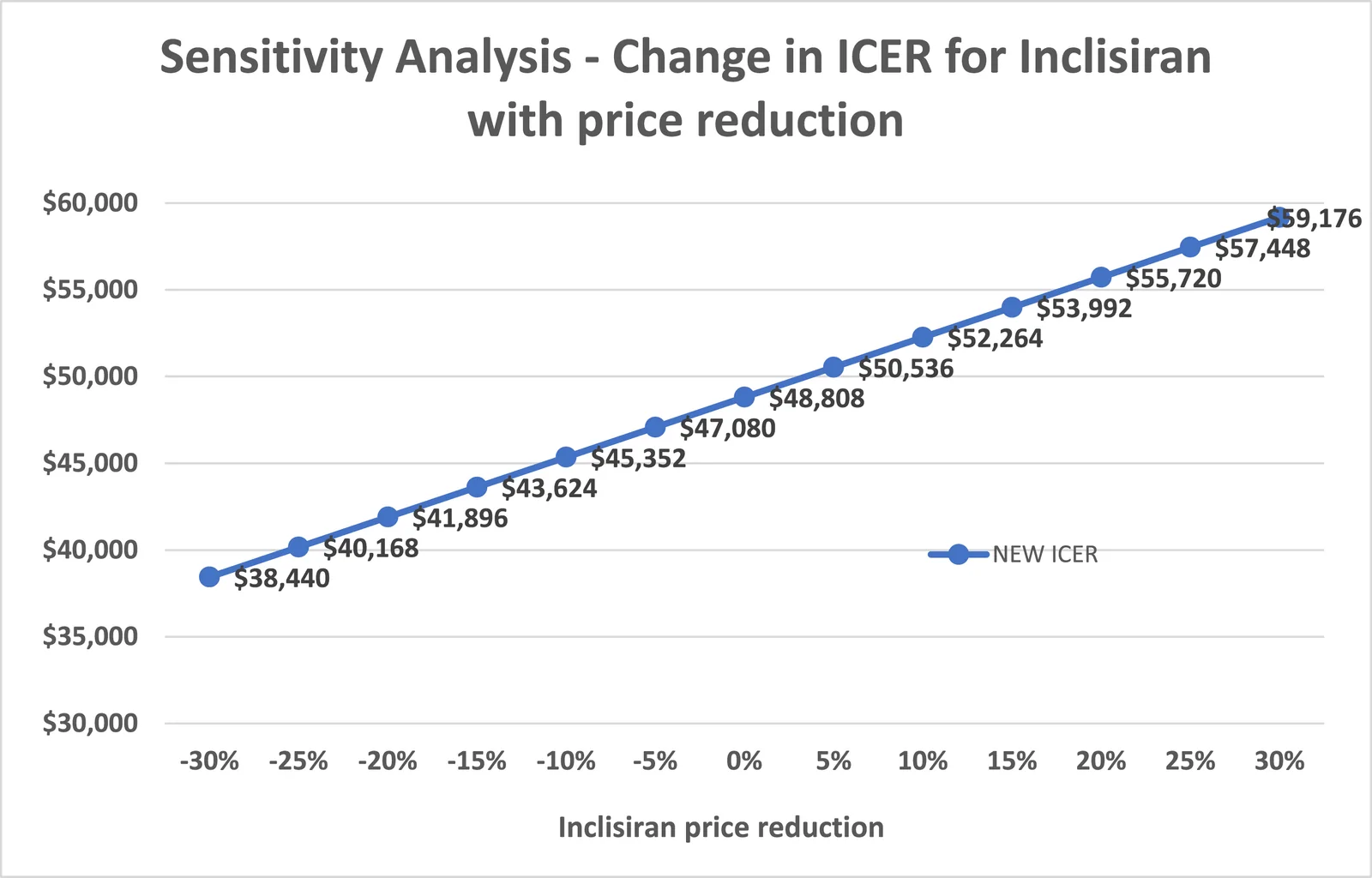
Sensitivity analysis – Change in ICER per QALY for Inclisiran with price reduction.

The addition of inclisiran to statins as first-line therapy in the target population was modelled to save 6401 non-fatal CVD events and gain 23,730 QALY's over 25 years at an additional cost of $1,158,188,332 (or $46,327,533/year) to the Australian healthcare system (Table 4). This would represent 0.32% of the total Australian governments' 2022 CVD expenditure [1]. The ICER was $48,808/QALY (Table 4). The WTPT of inclisiran dropped under the hypothetical Australian government PBAC $50,000AUD threshold at the 10th year, making it incrementally more cost-effective for the following 11 years thereafter (Fig. 2). Linear inclisiran price reduction linearly reduced the ICER (Fig. 3).

**Table 4:** – Markov model results for novel strategy of early-access Inclisiran use in the target population after 25 years.

Outcomes	Inclisiran + standard therapy	Standard Therapy	Difference
Total non-fatal CVD events	79,051	72,649	(6401)
Total fatal CVD events	6255	5185	1070
Total QALY's gained	154,495	130,765	(23,730)
Total Cost	$3,415,398,727	$2,644,042,927	$1,158,188,332 (or $46,327,533/year)
ICER	-	-	**$48,808**

### 3.2 Sensitivity analysis

**Fig. 4:**
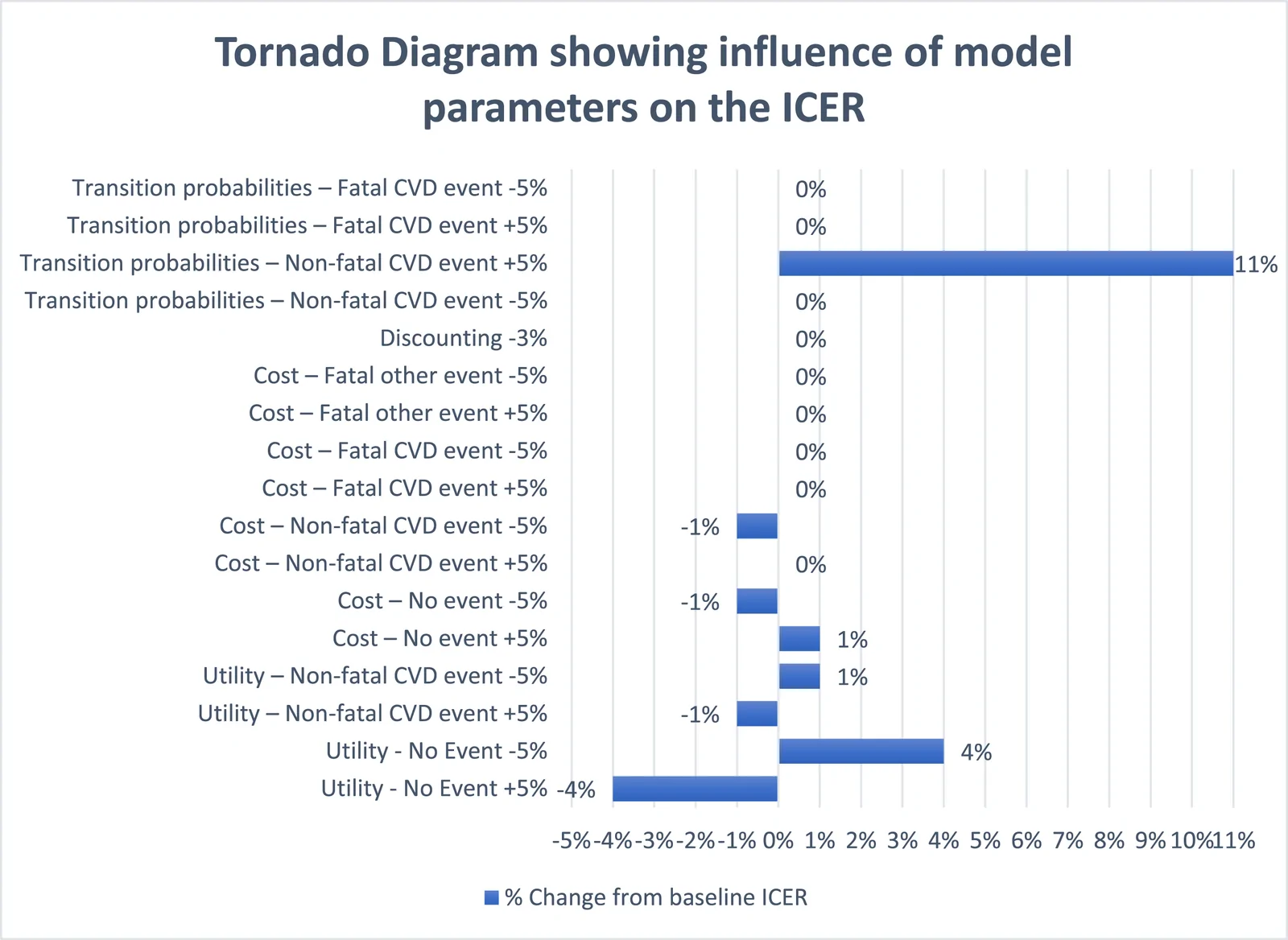
Tornado diagram showing influence of model parameters on the ICER.

Deterministic sensitivity analysis was performed on key model inputs to assess their influence on the primary outcome (Table 5, Fig. 4). Unless otherwise specified, parameters were varied by ±5%, with the discount rate additionally examined at 3%. No ±5% variation altered the ICER by more than 5%, except for recurrent non-fatal CVD event rates, which increased the ICER by 11%. Utility for the no-event health state altered the ICER by ±4%, while all other parameters changed the ICER by ≤ 1%. Broader (±20%) scenario analyses confirmed that the ICER was most sensitive to recurrent non-fatal CVD event rates, health-state utility estimates, and inclisiran cost, with some scenarios crossing commonly cited Australian willingness-to-pay thresholds (Appendix 3). Probabilistic sensitivity analysis was not performed because robust variance estimates were unavailable for several key model parameters. Future studies should incorporate probabilistic sensitivity analysis once robust parameter uncertainty estimates become available to better characterise decision uncertainty.

**Table 5:** Deterministic sensitivity analysis data. Model assumptions challenged via multiple new scenarios to establish their effect on the ICER.

	New Value	New ICER per QALY	% Change from baseline ICER
Utility - No Event	+5%	$46,848	−4%
	−5%	$50,934	4%
Utility – Non-fatal CVD event	+5%	$48,352	−1%
	−5%	$49,272	1%
Cost – No event	+5%	$49,058	1%
	−5%	$48,557	−1%
Cost – Non-fatal CVD event	+5%	$48,693	0%
	−5%	$48,516	−1%
Cost – Fatal CVD event	+5%	$48,973	0%
	−5%	$48,643	0%
Cost – Fatal other event	+5%	$48,879	0%
	−5%	$48,797	0%
Discounting	Reduced to 3%	$48,767	0%
Transition probabilities – Non-fatal CVD event	−5%	$48,939	0%
	+5%	$54,166	11%
Transition probabilities – Fatal CVD event	−5%	$48,577	0%
	+5%	$49,044	0%

## 4 Discussion

In this analysis, earlier addition of inclisiran to statin therapy in Aboriginal and Torres Strait Islander Australians with established CVD and hypercholesterolaemia was projected to prevent 6401 non-fatal cardiovascular events and gain 23,730 QALYs over 25 years, with an ICER of AUD48,808/QALY. These findings suggest that earlier access to inclisiran may represent a clinically meaningful and potentially cost-effective strategy in a population with substantial unmet cardiovascular need.

With Australian healthcare expenditure rising due to increasing age, frailty, comorbidities and complexity of care, cost-effective interventions have never been more important. Targeting therapies toward populations at highest absolute cardiovascular risk may improve both clinical benefit and economic efficiency [18].

Inclisiran has demonstrated LDL-C lowering efficacy with a favourable tolerability profile in clinical trials and real-world studies to date [4,8,9]. However, its integration into routine practice remains influenced by two key considerations. Firstly, despite patient-level pooled analyses of the ORION-9/10/11 trials trend towards MACE reduction, no long-term dedicated MACE-specific outcomes data exist. Cost-effectiveness analyses published in the literature to-date, use pooled CVD outcome data from the above trials [6,14,[19], [20], [21]]. The pending ORION-4 and VICTORION-2P trials aim to provide these data. Secondly, inclisiran remains a high-cost intervention costing over 40 times more than statins in some cases. Drug acquisition cost is a major determinant of cost-effectiveness, as reflected in prior international evaluations reporting ICERs of USD 157,000/QALY and EUR 72,239/QALY in US and Irish settings, respectively [19,22].

To our knowledge, the only prior Australian cost-effectiveness analysis of inclisiran was reported by Kam et al. using a similar Markov framework [14]. That study did not specifically examine Aboriginal and Torres Strait Islander Australians. Their higher ICER (AUD 125,732/QALY) compared with our findings may reflect differences in baseline cardiovascular risk, population selection, model assumptions, and outcome metrics. Our results support the broader principle that interventions may appear more cost-effective when targeted to populations at higher absolute cardiovascular risk. Apart from our study, up to early 2025 only one study concluded that inclisiran was cost-effective. The UK's National Institute for Health and Care Excellence (NICE) calculated an ICER of <£20,000/QALY. Critically, however, the cost of inclisiran was reduced for this assessment but was not disclosed [20]. Fundamentally, the price of inclisiran would appear to highly elastic, and directly proportional to cost-effectiveness.

Furthermore, our modelling, as has other global models, demonstrates the that earlier inclisiran use, and over longer durations, improves its’ clinical benefit and cost-efficacy [17].

Inclisiran also has practical characteristics that may differentiate it from oral lipid-lowering therapies, particularly its twice-yearly administration by healthcare professionals. This schedule may offer opportunities to integrate treatment delivery with structured secondary prevention follow-up, medication review, and cardiovascular risk reassessment, a concept explored by Nelson et al. [21]. imilar service models have been explored in other chronic disease programs for geographically dispersed and high-risk populations. Whether such approaches improve adherence, persistence, or outcomes in Aboriginal and Torres Strait Islander Australians requires prospective evaluation.

Potential implementation framework could include culturally safe identification of eligible patients during hospitalisation or outpatient review [23], linkage to follow-up registries, optimisation of secondary prevention therapies, and coordinated recall systems including automated messaging and/or transport assistance for 6-monthly review and inclisiran administration [23,24] Partnerships with Aboriginal Community Controlled Health Organisations may be particularly important to maximise accessibility, continuity, and culturally responsive care [23,24]. Furthermore, regular practitioner visitations allow for assessment of ongoing CVD risk and aggressive risk factor modification [25,26]. In rural and remote settings, expanded access pathways may further improve equitable service delivery [27]. These strategies are currently hypothetical and should be tested in implementation studies before wider adoption.

Equity and clinical need are also considered by the PBAC in funding decisions [4]. How health equity gains should be incorporated within conventional willingness-to-pay frameworks remains uncertain and warrants further policy and methodological work. Equity-weighting frameworks propose that health gains achieved in disadvantaged populations may warrant greater societal value. Consequently, interventions with ICERs close to conventional willingness-to-pay thresholds may be viewed more favourably when targeted toward Aboriginal and Torres Strait Islander Australians. Given the disproportionate CVD burden that Aboriginal and Torres Strait Islander Australians carry, and the structural barriers to healthcare that exists in the Australian system preventing equal healthcare use and access [23], there is clinical need for equitable solutions. These findings suggest that, under certain willingness-to-pay thresholds, inclisiran may represent a potentially equitable and clinically effective addition to secondary prevention strategies. However, reimbursement decisions would also require consideration of affordability, budget impact, feasibility, and competing priorities.

This study has several strengths, including use of the best available Australian epidemiological data, transparent modelling assumptions, and a focus on a population with substantial unmet cardiovascular need. Nevertheless, several limitations should be acknowledged. No real-world data currently exist regarding inclisiran uptake, adherence, or effectiveness in Aboriginal and Torres Strait Islander Australians. By assuming equal adherence across treatment arms, the model intentionally isolated pharmacological treatment effects; however, the twice-yearly administration of inclisiran may improve treatment persistence relative to daily oral therapies, potentially underestimating its real-world effectiveness and cost-effectiveness. Long-term cardiovascular outcomes were extrapolated from pooled ORION-9/10/11 trial data in a different population and should therefore be interpreted as modelled rather than observed outcomes. As with all Markov models, simplification of clinical pathways and the memoryless structure may not fully capture recurrent risk trajectories over time. Although deterministic and broader scenario analyses explored uncertainty in key model inputs, probabilistic sensitivity analysis could not be performed because robust parameter uncertainty estimates were unavailable. This is particularly relevant given the base-case ICER lies close to commonly cited Australian willingness-to-pay thresholds, meaning relatively small changes in key assumptions may influence conclusions regarding cost-effectiveness. Accordingly, these findings should be interpreted as exploratory until future studies incorporating robust parameter distributions, population-specific transition probabilities and utility estimates, and dedicated cardiovascular outcomes data permit formal probabilistic sensitivity analysis and more definitive economic evaluation.

## 5 Conclusions

In this modelled analysis, earlier addition of inclisiran to statin therapy for Aboriginal and Torres Strait Islander Australians with established CVD and hypercholesterolaemia was associated with projected reductions in cardiovascular burden, including an estimated 6401 fewer non-fatal cardiovascular events and 23,730 QALYs gained over 25 years. At an incremental cost of AUD 1.16 billion (0.32% of annual Australian government CVD expenditure), the ICER of AUD 48,808 per QALY gained was within the range of commonly referenced Australian willingness-to-pay thresholds. Cost-effectiveness improved with lower inclisiran pricing and earlier treatment initiation. These findings suggest that earlier, equity-focused access to inclisiran may represent a potentially cost-effective secondary prevention strategy and may help inform future reimbursement, guideline, and cardiovascular prevention policy decisions in Australia.

## CRediT authorship contribution statement

**Satyen Hargovan:** Conceptualization, Data curation, Formal analysis, Investigation, Methodology, Project administration, Resources, Validation, Visualization, Writing – original draft. **Nadine Hunt:** Conceptualization, Methodology, Project administration, Supervision, Writing – review & editing. **Hara Kostakis:** Conceptualization, Methodology, Project administration, Supervision, Writing – review & editing. **Clara K. Chow:** Conceptualization, Methodology, Project administration, Supervision, Writing – review & editing.
