# Supplementary material for: Cost-effectiveness of early inclisiran for the secondary prevention of cardiovascular disease in Aboriginal and Torres Strait Islander Australians: A Markov modelling analysis
Source: Int J Cardiol Cardiovasc Risk Prev. 2026 Aug 7;30:200687. doi: 10.1016/j.ijcrp.2026.200687 (PMC13498953; doi:10.1016/j.ijcrp.2026.200687)
Supplement: Supplementary file 1 [file mmc1.docx]

Appendix 1: Data, formulas and methodologies used to construct the Markov model. MACE = major adverse cardiovascular events, DALY = Disability-adjusted life year, HR = hazard ratio, CVD = cardiovascular disease

| **Intervention** | **Outcome category** | **Outcome measure** | **Methodology (yearly)** |
| --- | --- | --- | --- |
| Inclisiran (pooled ORION 9/10/11 trials) | Outcomes | No event | 100% – (non-fatal CVD event + fatal CVD event + fatal other event). |
|  |  | Non-fatal CVD event | 114/1833 non-fatal CVD events in the Inclisiran arm vs. 157/1827 in Placebo arm = absolute risk reduction of 2.37% |
|  |  | Fatal CVD event | 17/1833 fatal CVD deaths in Inclisiran arm vs. 15/1827 in Placebo arm = absolute risk increase of 0.11% |
|  |  | Fatal other event | Assumed same as control arm to remove this potential confounder. |
|  | QALY's | No event | 12,180 at-risk x 0.8 QALY |
|  |  | Non-fatal CVD event | 12,180 at-risk x 0.7 QALY |
|  |  | Fatal CVD event | 0 |
|  |  | Fatal other event | 0 |
|  | Costs | Statin + Inclisiran | $223 + 3699 (annual cost) |
|  |  | No event | $4,878 |
|  |  | Non-fatal CVD event | $21,943 |
|  |  | Fatal CVD event | $76,927 |
|  |  | Fatal Other event | $76,927 |
| Standard Therapy | Outcomes | No event | 100% – (non-fatal CVD event + fatal CVD event + fatal other event). |
|  |  | Non-fatal CVD event | 5010 CVD-related hospitalisations – the 334 annual deaths in Aboriginal and Torres Strait Islander Australians with CVD and hypercholesterolaemia per the Australian Institute for Health and Wellbeing = 4,676 total non-fatal CVD events. |
|  |  | Fatal CVD event | 334 annual deaths in Aboriginal and Torres Strait Islander Australians with CVD and hypercholesterolaemia per the Australian Institute for Health and Wellbeing |
|  |  | Fatal other event | Age-adjusted Indigenous Australian mortality rate for 55-74 years of age (to match the cohort age of the ORION trials) = 2,151/100,000 or 2.151%. 28.65% of deaths in this cohort are due to CVD, so 71.65% of 2.151 = 1.54% |
|  | QALY's | No event | 12,180 at-risk x 0.8 QALY |
|  |  | Non-fatal CVD event | 12,180 at-risk x 0.7 QALY |
|  |  | Fatal CVD event | 0 |
|  |  | Fatal other event | 0 |
|  | Costs | Statin only | $223 |
|  |  | No event | $4,878 |
|  |  | Non-fatal CVD event | $21,943 |
|  |  | Fatal CVD event | $76,927 |
|  |  | Fatal other event | $76,927 |
