# Supplementary material for: Cost-effectiveness of early inclisiran for the secondary prevention of cardiovascular disease in Aboriginal and Torres Strait Islander Australians: A Markov modelling analysis
Source: Int J Cardiol Cardiovasc Risk Prev. 2026 Aug 7;30:200687. doi: 10.1016/j.ijcrp.2026.200687 (PMC13498953; doi:10.1016/j.ijcrp.2026.200687)
Supplement: Supplementary file 2 [file mmc2.docx]

AdViSHE

Assessment of the Validation Status of Health-Economic decision models

AdViSHE is a questionnaire that modellers can complete to report on the efforts performed to improve the validation status of their health-economic (HE) decision model. It is not intended to replace validation by model users but rather to inform the direction of validation efforts and to provide a baseline for replication of the results. In addition to using it after a model is finished, the modellers can use AdViSHE to guide validation efforts during the modelling process.

The modellers are asked to comment on the validation efforts performed while building the underlying HE decision model and afterwards. Many of the questions simply refer to the model documentation. AdViSHE is divided into five parts, each covering an aspect of validation:

- Part A: Validation of the conceptual model (2 questions)
- Part B: Input data validation (2 questions)
- Part C: Validation of the computerized model (4 questions)
- Part D: Operational validation (4 questions)
- Part E: Other validation techniques (1 question)

No final validation score is calculated, as the assessment of the answers and the overall validation effort is left to the model users. It is assumed that the model has been built according to prevailing modelling and reporting guidelines. For instance, the model builders would presumably adhere to the ISPOR-SMDM^†^ Modeling Good Research Practices (Caro et al., 2010) and/or CHEERS^†^ Statement (Husereau et al., 2013). Some questions may not be applicable to a particular model. If this is the case, the model builder should take the opt-out option and provide a justification of why this item is not deemed applicable.

Part A: Validation of the conceptual model (2 questions)

Part A discusses techniques for validating the conceptual model. A conceptual model describes the underlying system (e.g., progression of disease) using a mathematical, logical, verbal, or graphical representation. Please indicate where the conceptual model and its underlying assumptions are described and justified.

Markov modelling is well described in the literature, as are markov models for CVD. Transition probabilities were taken from a brief targeted literature review of meta-analyses of RCT’s. Utilities, costs and discounting were described in our methodology. These are similar to other markov models using CVD.

**A1/ Face validity testing (conceptual model):** Have experts been asked to judge the appropriateness of the conceptual model?

If yes, please provide information on the following aspects:

- Who are these experts?
- What is your justification for considering them experts?
- To what extent do they agree that the conceptual model is appropriate?

If no, please indicate why not.

Markov modelling is well-described in the literature and has been widely externally validated. Whilst values in our markov model have undergone markov model testing previously (ie, paper by Kumar et al, 2018, entitled “The cost-effectiveness of PCSK9 inhibitors - The Australian healthcare perspective” uses FOURIER values for Evolocumab in an Australian population) but the model as a whole has not. This is the first paper to model an Indigenous Australian secondary prevention CVD cohort, and that may explain why.

Aspects to judge include: appropriateness to represent the underlying clinical process/disease (disease stages, physiological processes, etc.); and appropriateness for economic evaluation (comparators, perspective, costs covered, etc.).

**A2/ Cross validity testing (conceptual model):** Has this model been compared to other conceptual models found in the literature or clinical textbooks?

If yes, please indicate where this comparison is reported.

If no, please indicate why not.

Comparison to other similar models in an Australian healthcare setting, such as the one referenced above, are found in our discussion.

Part B: Input data validation (2 questions)

Part B discusses techniques to validate the data serving as input in the model. These techniques are applicable to all types of models commonly used in HE modelling.

Please indicate where the description and justification of the following aspects are given:

- search strategy;
- data sources, including descriptive statistics;
- reasons for inclusion of these data sources;
- reasons for exclusion of other available data sources;
- assumptions that have been made to assign values to parameters for which no data was available;
- distributions and parameters to represent uncertainty;
- data adjustments: mathematical transformations (e.g., logarithms, squares); treatment of outliers; treatment of missing data; data synthesis (indirect treatment comparison, network meta-analysis); calibration; etc.

These are all given in our methodology.

**B1/ Face validity testing (input data):** Have experts been asked to judge the appropriateness of the input data?

If yes, please provide information on the following aspects:

- Who are these experts?
- What is your justification for considering them experts?
- To what extent do they agree that appropriate data has been used?

If no, please indicate why not.

Yes, they agree it is appropriate data. The experts are Hara Kostakis, an academic in economic evaluation with the London School of Hygiene and Tropical Medicine. Also Dr. Clara Chow, Professor of Medicine and Consultant Cardiologist, Sydney, NSW, Australia. She has a PhD in Medicine from the University of Sydney and completed a postdoc in Cardiovascular epidemiology and clinical trials at McMaster University, Canada. Clara holds a Career Development Fellowship of the NHMRC co-funded by the National Heart Foundation and has over 100 publications.

Aspects to judge may include but are not limited to: potential for bias; generalizability to the target population; availability of alternative data sources; any adjustments made to the data.

**B2/ Model fit testing:** When input parameters are based on regression models, have statistical tests been performed?

If yes, please indicate where the description, the justification and the outcomes of these tests are reported.

If no, please indicate why not.

No input paramaters are based on regression models. Results are presented with confidence intervals and p-values where able.

Examples of regression models include but are not limited to: disease progression based on survival curves; risk profiles using regression analysis on a cohort; local cost estimates based on multi-level models; meta-regression; quality-of-life weights estimated using discrete choice analysis; mapping of disease-specific quality-of-life weights to utility values.

Examples of tests include but are not limited to: comparing model fit parameters (R^2^, Akaike information criterion (AIC), Bayesian information criterion (BIC)); comparing alternative model specifications (covariates, distributional assumptions); comparing alternative distributions for survival curves (Weibull, lognormal, logit); testing the numerical stability of the outcomes (sufficient number of iterations); testing the convergence of the regression model; visually testing model fit and/or regression residuals.

Part C: Validation of the computerized model (4 questions)

Part C discusses various techniques for validating the model as it is implemented in a software program. If there are any differences between the conceptual model (Part A) and the final computerized model, please indicate where these differences are reported and justified.

No differences.

**C1/ External review:** Has the computerized model been examined by modelling experts?

If yes, please provide information on the following aspects:

- Who are these experts?
- What is your justification for considering them experts?
- Can these experts be qualified as independent?
- Please indicate where the results of this review are reported, including a discussion of any unresolved issues.

If no, please indicate why not.

Yes, it has been examined by the experts listed in B1.

Aspects to judge may include but are not limited to: absence of apparent bugs; logical code structure optimized for speed and accuracy; appropriate translation of the conceptual model.

**C2/ Extreme value testing:** Has the model been run for specific, extreme sets of parameter values in order to detect any coding errors?

If yes, please indicate where these tests and their outcomes are reported.

If no, please indicate why not.

High and low parameters, +/- 50% of the original value, have been run internally for each outcome measure

Examples include but are not limited to: zero and extremely high (background) mortality; extremely beneficial, extremely detrimental, or no treatment effect; zero or extremely high treatment or healthcare costs.

**C3/ Testing of traces:** Have patients been tracked through the model to determine whether its logic is correct?

If yes, please indicate where these tests and their outcomes are reported.

If no, please indicate why not.

Yes, patient numbers across the years and different health states add up to the initial value.

In cohort models, this would involve listing the number of patients in each disease stage at one, several, or all time points (e.g., Markov traces). In individual patient simulation models, this would involve following several patients throughout their natural disease progression.

**C4/ Unit testing:** Have individual sub-modules of the computerized model been tested?

If yes, please provide information on the following aspects:

- Was a protocol that describes the tests, criteria, and acceptance norms defined beforehand?
- Please indicate where these tests and their outcomes are reported.

If no, please indicate why not.

Formulas have been tested, parameters have been altered and there are no redundencies.

Examples include but are not limited to: turning sub-modules of the program on and off; altering global parameters; testing messages (e.g., warning against illegal or illogical inputs), drop-down menus, named areas, switches, labelling, formulas and macros; removing redundant elements.

Part D: Operational validation (4 questions)

Part D discusses techniques used to validate the model outcomes.

**D1/ Face validity testing (model outcomes):** Have experts been asked to judge the appropriateness of the model outcomes?

If yes, please provide information on the following aspects:

- Who are these experts?
- What is your justification for considering them experts?
- To what extent did they conclude that the model outcomes are reasonable?

If no, please indicate why not.

Yes, see experts listed in B1. They deem outcomes appropriate as also reported widely throughout the literature.

Outcomes may include but are not limited to: (quality-adjusted) life years; deaths; hospitalizations; total costs.

**D2/ Cross validation testing (model outcomes):** Have the model outcomes been compared to the outcomes of other models that address similar problems?

If yes, please provide information on the following aspects:

- Are these comparisons based on published outcomes only, or did you have access to the alternative model?
- Can the differences in outcomes between your model and other models be explained?
- Please indicate where this comparison is reported, including a discussion of the comparability with your model.

If no, please indicate why not.

Similar models have been compared to ours, and are found in our discussion. See papers on Australian CVD cost-effectiveness by Zomer and Liew et al (can be found in our reference list).

Other models may include models that describe the same disease, the same intervention, and/or the same population.

**D3/ Validation against outcomes using alternative input data:** Have the model outcomes been compared to the outcomes obtained when using alternative input data?

If yes, please indicate where these tests and their outcomes are reported.

If no, please indicate why not.

Few alternative data exist given that PCSK9-pharmacotherapies are relatively novel. Only few papers describe clinical endpoints that are able to be used for outcome modelling.

Alternative input data can be obtained by using different literature sources or datasets, but can also be constructed by splitting the original data set in two parts, and using one part to calculate the model outcomes and the other part to validate against.

**D4/ Validation against empirical data:** Have the model outcomes been compared to empirical data?

If yes, please provide information on the following aspects:

- Are these comparisons based on summary statistics, or patient-level datasets?
- Have you been able to explain any difference between the model outcomes and empirical data?
- Please indicate where this comparison is reported.

If no, please indicate why not.

**D4.A/** Comparison against the data sources on which the model is based (dependent validation).

The markov model outcomes were compared against known data including annual CVD deaths, annual fatal other events and annual non-fatal CVD events. This is found in our results and discussion.

**D4.B/** Comparison against a data source that was not used to build the model (independent validation).

There are no markov models that have used a secondary prevention CVD cohort of Indigenous Australians with dyslipidaemia. Data sources are taken from large Australian national governmental official statistics.

Part E: Other validation techniques (1 question)

**E1/ Other validation techniques:** Have any other validation techniques been performed?

If yes, indicate where the application and outcomes are reported, or else provide a short summary here.

Models walked through with experts listed in B1.

Examples of other validation techniques: structured “walk-throughs” (guiding others through the conceptual model or computerized program step-by-step); naïve benchmarking (“back-of-the-envelope” calculations); heterogeneity tests; double programming (two model developers program components independently and/or the model is programmed in two different software packages to determine if the same results are obtained).
