# Supplementary material for: Cost-effectiveness of early inclisiran for the secondary prevention of cardiovascular disease in Aboriginal and Torres Strait Islander Australians: A Markov modelling analysis
Source: Int J Cardiol Cardiovasc Risk Prev. 2026 Aug 7;30:200687. doi: 10.1016/j.ijcrp.2026.200687 (PMC13498953; doi:10.1016/j.ijcrp.2026.200687)
Supplement: Supplementary file 3 [file mmc3.docx]

|  | **New Value** | **New ICER per QALY** | **% Change from baseline ICER** |
| --- | --- | --- | --- |
| **Cost – Inclisiran** | +20% | $55,310 | +13% |
|  | -20% | $42,278 | -13% |
| **Utility – Non-fatal CVD event** | +20% | $33,822 | +30% |
|  | -20% | $87,636 | -79% |
| **Transition probabilities – Non-fatal CVD event** | +20% | $35,650 | +26% |
|  | -20% | $63,542 | -30% |

*Appendix 3: Broader scenario analysis +/- 20% for key model drivers*
